# Complete genome sequences of three *Geobacillus stearothermophilus* strains isolated from a Korean hot spring

**DOI:** 10.1128/mra.00940-25

**Published:** 2025-12-15

**Authors:** Dariimaa Ganbat, Jae-Yoon Sung, Seong-Bo Kim, Sondor Ganbat, Mi Hwa Park, Dong-Woo Lee, Sang-Jae Lee

**Affiliations:** 1 Department of Bioscience and Research Center for Extremophiles and Marine Microbiology, Silla University 65486, Busan, South Korea; 2 Department of Biotechnology, Yonsei University, Seoul, South Korea; 3 Bio-Living Engineering Major, Global Leaders College, Yonsei University, Seoul, South Korea; 4 Department of Food and Nutrition, College of Medical and Life Science, Silla University 65486, Busan, South Korea; California State University San Marcos, San Marcos, California, USA

**Keywords:** *Geobacillus stearothermophilus*, thermophile, genome, plasmid, hot spring

## Abstract

We present the complete genome sequences of three *Geobacillus stearothermophilus* strains—EF60063, EF60133, and EF60134—isolated from a Korean hot spring. S8 family serine peptidases and M4 family metallopeptidases were identified in all strains. Strain EF60063 harbors three plasmids. These findings advance understanding of the genus *Geobacillus*.

## ANNOUNCEMENT

**Fig 1:**
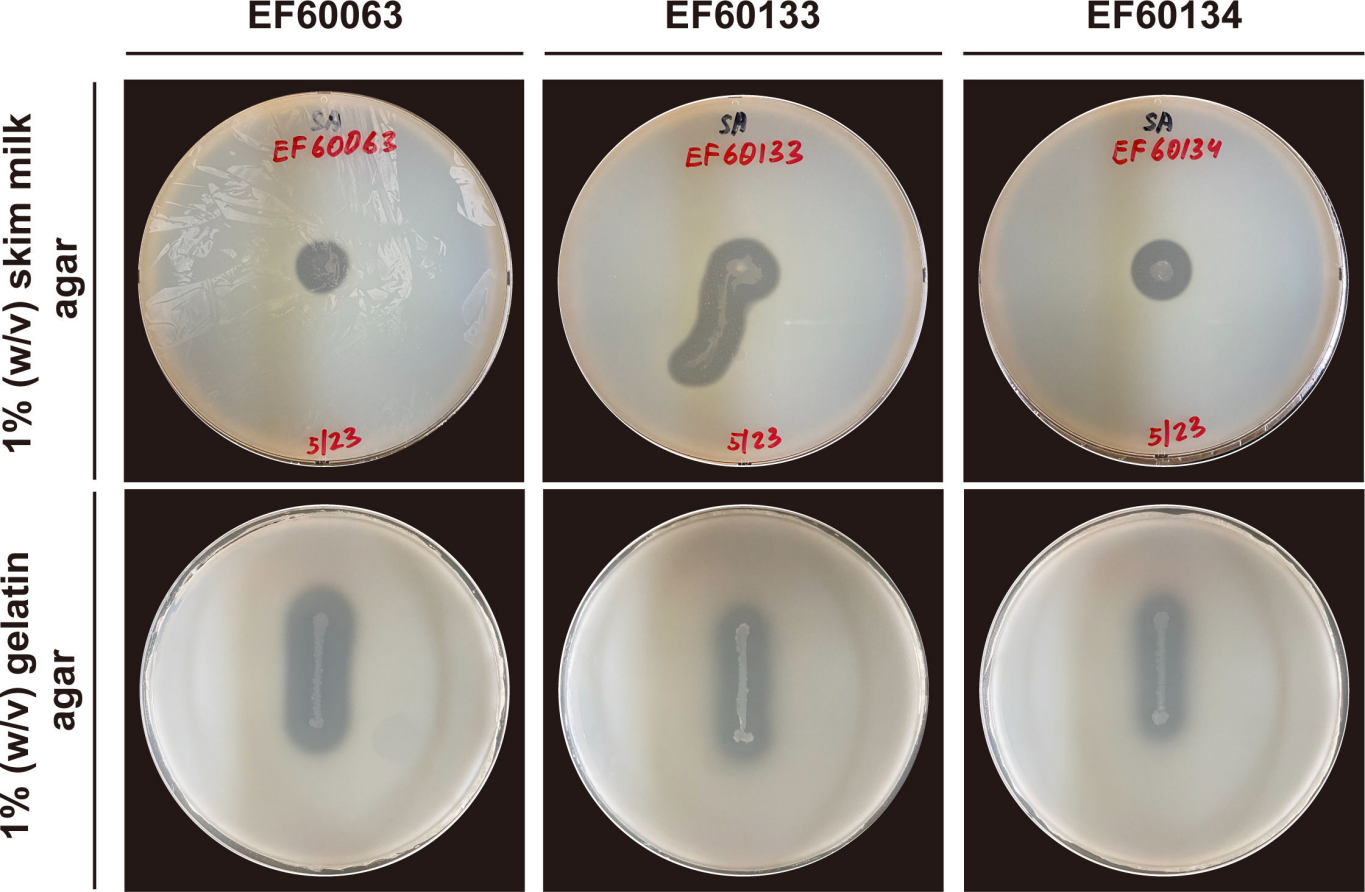
Extracellular protease activity of *Geobacillus stearothermophilus* strains EF60063, EF60133, and EF60134 on 1% (wt/vol) skim milk agar (upper) and 1% (wt/vol) gelatin agar (lower) plates after incubation at 55°C for 72 h.

*Geobacillus* species are recognized for their metabolic versatility and enzymatic capabilities, widely utilized in various industrial and biotechnological applications, and remain promising candidates for research (1–3). We isolated multiple thermophilic strains from Korean hot springs (4), assessed their enzymatic activities, and identified several *Geobacillus* isolates exhibiting proteolytic activity; among these, three strains were selected for whole-genome sequencing (Fig. 1). These strains, EF60063, EF60133, and EF60134, were isolated from a Mungang Hot Spring (26.4–28.3°C, pH 8.9–9.1) water sample that was collected on 18 January 2017 in South Korea (36°53′15.4″ N 127°57′20.9″ E). 1 mL of water was serially diluted in 0.85% saline, and was spread on marine agar plates (BD Difco 2216). Plates were incubated at 60°C for 1 week, and the obtained isolates were cryopreserved in 5% (vol/vol) DMSO at −80°C. Strains were identified by amplifying the 16S rRNA gene using BioFACT S-Taq DNA polymerase with universal primers 27F and 1492R, and taxonomic assignment was confirmed via NCBI BLASTN v2.8.0+ (Table 1).

**TABLE 1:** Summary of quality assessments for bacterial strains sequenced in this study

Isolate	Closely related species	Identity (%)	BioSample accession number	SRA accession number (O/P: ONT/PacBio, I: Illumina)	Total number of raw reads	Read *N*_50_ (bp)	GenBank accession number	No. of contigs	Type of contig	Genome size (bp)	GC content (%)	Coverage (×)	Total genes	Protein-coding genes
EF60063	*Geobacillus stearothermophilus* BGSC 9A20 (AY608928)	99.60	SAMN35768708	P: SRR35022333	212,884	6,075	CP128454	4	Chromosome	3,435,864	52.5	251.0	3,677	3,353
							CP128455		pEF60063-1	65,200	40.5			
				I: SRR35022332	10,348,816		CP128456		pEF60063-2	37,039	43.0			
							CP128457		pEF60063-3	2,180	48.0			
EF60133	*Geobacillus stearothermophilus* BGSC 9A20 (AY608928)	99.87	SAMN35768736	O: SRR35048013	1,091,881	4,622	CP128458	1	Chromosome	3,654,319	52.0	732.0	3,714	3,426
				I: SRR35030277	14,697,890									
EF60134	*Geobacillus stearothermophilus* BGSC 9A20 (AY608928)	99.93	SAMN35768739	P: SRR35022364	321,844	8,152	CP128459	1	Chromosome	3,602,581	52.0	529.9	3,744	3,409

Following the culture conditions detailed in our previous study (5), strains were grown at 60°C in mLB medium, and genomic DNA (gDNA) was extracted using the Wizard Genomic DNA Purification Kit (Promega).

The Oxford Nanopore Technologies (ONT) MinION platform was used to generate long reads for EF60133. gDNA was sequenced on an ONT MinION (R10.4, FLO-MIN112) using the SQK-LSK112.24 ligation kit with NEBNext Companion Module for library preparation, without DNA shearing and size selection. Base calling was performed with Guppy v6.0.6 and read quality assessed using NanoStat v1.4.0 (6). Adapters auto-trimmed during base calling were verified using FastQC v0.12.0 (7). Simultaneously, Illumina sequencing was performed on a NovaSeq 6000 (paired-end 2 × 150 bp), with libraries prepared using the TruSeq Nano DNA Sample Prep Kit. Reads were trimmed using Trimmomatic v0.39 (8) and assessed with FastQC v0.12.0. The genome was assembled with Flye v2.9.2 (9), and the consensus sequence was polished using bcftools v1.20 (10) (parameters “–minDP 5 –minQ 30”) based on variant calling results.

For EF60063, sequencing was performed on the PacBio Sequel platform using the Sequel Sequencing Kit 3.0, following the standard workflow. The sequencing library was prepared using the SMRTbell Express Template Prep Kit 2.0 (Pacific Biosciences). DNA was sheared by g-TUBE (Covaris), size-selected (15–20 kb) with AMPure PB beads, and reads were quality-filtered with a Q20 threshold. Additionally, Illumina sequencing was performed as described above. The genome was assembled with the HGAP v4.0 (11), followed by polishing to refine the consensus.

Finally, EF60134 was sequenced on the PacBio Sequel platform as described above and assembled using HGAP v4.0.

Circular assemblies were processed with Circlator v1.5.5 and oriented to begin at the *dnaA* gene (12). Assemblies were annotated using the NCBI PGAP v.6.5 (13). S8 family serine peptidases and M4 family metallopeptidases were identified in all strains. Default parameters were used unless noted otherwise.

## Data Availability

The WGS projects for the strains EF60063, EF60133, and EF60134 have been deposited in GenBank under BioProject number PRJNA983507. Further details and accession numbers are listed in Table 1.
